# Clocking immunity: circadian modulation of NK cells and emerging timing perspectives

**DOI:** 10.3389/fimmu.2026.1739723

**Published:** 2026-06-04

**Authors:** Kun Liu, Xuanying Lv, Wen Wen

**Affiliations:** Third Affiliated Hospital of Naval Medical University (Second Military Medical University), National Center for Liver Cancer, Shanghai, China

**Keywords:** chronotherapy, circadian rhythms, degranulation, glucocorticoids, natural killer cells, neuroendocrine regulation, shift work, sleep deprivation

## Abstract

Natural killer (NK) cells show day–night variation in both number and effector function. We compile findings from human cohorts, animal models, and cell studies on how circadian timing shapes NK biology. Evidence spans daily changes in counts and readouts such as degranulation and IFN-γ; links to core clock modules (PER1/2, NFIL3/E4BP4, STRA13); and neuroendocrine inputs (sympathetic tone, glucocorticoids, melatonin). Acute sleep loss can transiently raise NK activity, whereas multi-day sleep deprivation or circadian misalignment lowers counts or function. Shift work studies and laboratory night-shift simulations show reductions in NK activity and phase-sensitive changes in transcriptional programs (e.g., AP-1/STAT), with effects amplified by irregular schedules and accumulated fatigue. Across cancer, depression, vitiligo, and infection, alterations are heterogeneous, often presenting as peak shifts or amplitude flattening rather than loss of rhythmicity. Photoperiod, season, age, and sex can modify these patterns. Current data support a circadian influence on NK biology, but the direction and magnitude of reported effects vary across species, sampling schedules, circadian phase definitions, tissue compartments, and NK-cell readouts. This heterogeneity underscores the need for longitudinal, high-frequency sampling with complementary continuous monitoring to define phase, amplitude, and stability across physiological and clinical contexts.

## 1 Introduction

Circadian rhythms refer to endogenous physiological and behavioral changes in organisms that manifest as approximately 24-hour cycles, under the regulatory control of intrinsic clock genes (1). These rhythmic processes are extensively distributed across diverse tissues and organs in mammals, and play a pivotal role in maintaining the body’s physiological homeostasis as well as modulating its adaptive capacity to environmental perturbations (2, 3). Specifically, within the immune system, the quantity, functional activity, and cytokine secretion profiles of immune cells display prominent circadian oscillations (4–7). These rhythmic variations contribute to immune homeostasis, as the timing of immune-cell activity can influence host susceptibility to infection, tumor-cell elimination, and immune surveillance (8, 9).

Natural killer (NK) cells, a pivotal constituent of the human innate immune system, exert a critical defensive role in safeguarding the organism against tumor cells, viral infections, and other pathogenic agents (10). In recent years, a growing body of research has supported the circadian modulation of immune function, including NK-cell number and activity. However, the direction and magnitude of these effects are not uniform across studies and may vary according to species, tissue compartment, sampling schedule, circadian phase definition, and the specific functional endpoint assessed. This rhythmicity is manifested not only in the quantity and functional activity of NK cells but also across multiple immune regulatory pathways, and maintains a close functional association with the organism’s neuroendocrine system (11).

In recent years, researchers have elucidated the intricate relationship between circadian rhythms and NK cell function, along with underlying molecular mechanisms governing this relationship. These mechanisms encompass reciprocal regulation between core clock genes (such as NFIL3, PER1, PER2) and the processes of NK cell development, activation, and functional execution, as well as the regulatory pathways through which the sympathetic nervous system and the hypothalamic-pituitary-adrenal (HPA) axis modulate immune rhythms via the release of neurotransmitters and hormonal mediators (12–14). These studies offer new perspectives on the dynamic regulation of the immune system and establish a crucial theoretical foundation for understanding the relationship between circadian rhythm disorders and disease onset and progression.

This review examines circadian rhythmicity in NK-cell number and function under physiological and pathological conditions. It also discusses how circadian disruption, sleep loss, shift work, and disease-associated neuroendocrine changes may alter NK-cell timing, redistribution, and effector readouts. Furthermore, this review will discuss how circadian rhythms shape the phase and amplitude of NK-cell number and effector functions via molecular clocks and the neuroendocrine-immune axis, resulting in oppositely directed phenotypes across different time windows. A central premise of this review is that circadian effects on NK cells should be interpreted in a phase-, context-, and readout-dependent manner. Apparent discrepancies across studies may reflect differences in species, tissue compartment, sampling schedule, exposure duration, disease stage, and whether NK-cell number, cytotoxicity, degranulation, cytokine production, or transcriptional programs were assessed. Therefore, we distinguish, where possible, between mechanistic evidence from experimental perturbation studies and associative findings from clinical or occupational cohorts. By distinguishing mechanistic evidence from associative clinical and occupational findings, this review provides an interpretive framework for NK-cell circadian biology and identifies evidence gaps that must be addressed before timing-based interventions can be translated into routine clinical or public health practice.

## 2 NK cells and circadian rhythms under physiological homeostasis

### 2.1 Circadian fluctuations in the quantity and function of NK cells and hormonal regulation

**Figure 1:**
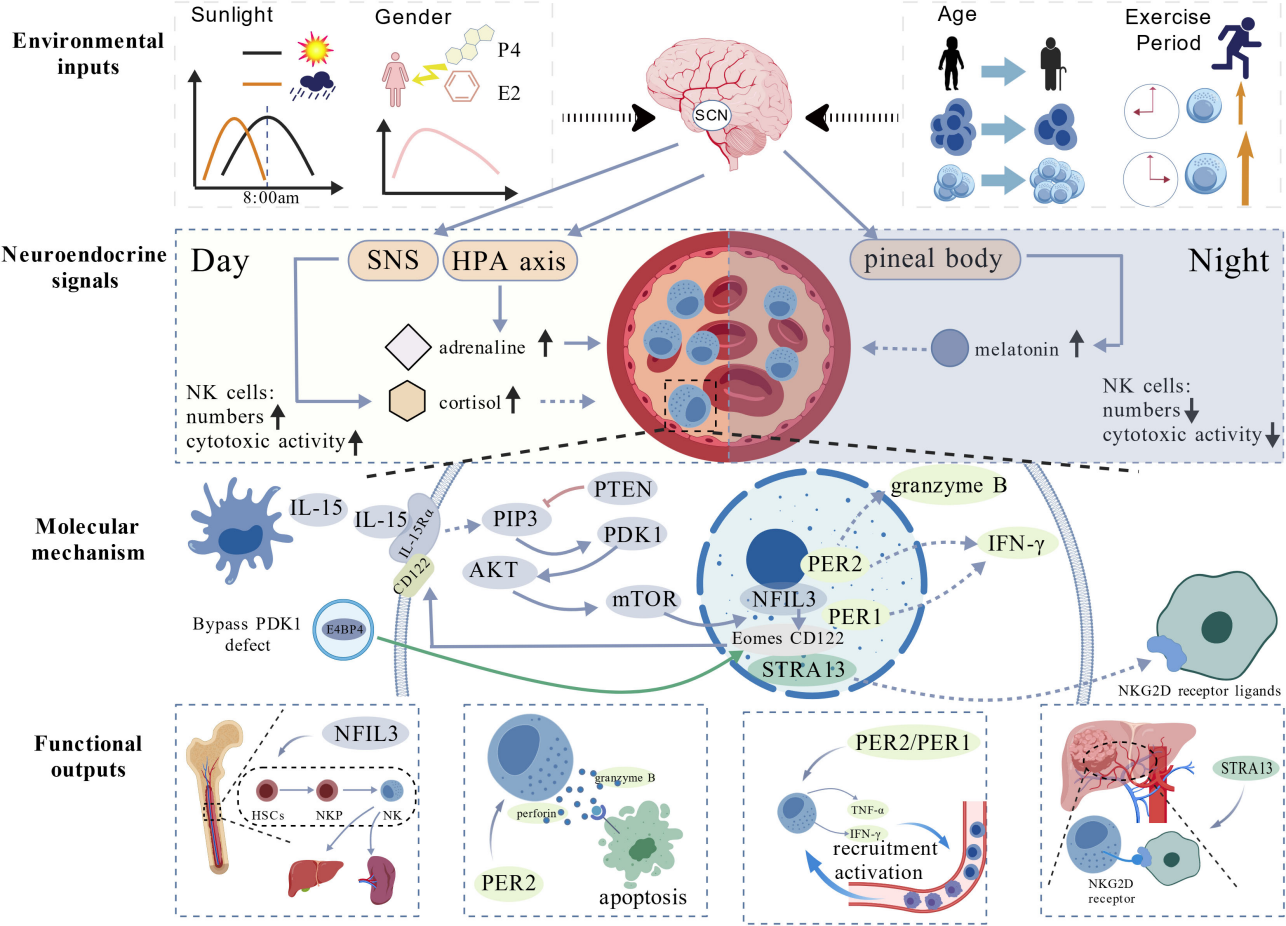
Environmental, physiological, and neuroendocrine regulation of circadian NK-cell function. Light exposure and host-related factors, including age, sex/gender-related differences, and exercise timing, provide timing-related inputs that are integrated by the suprachiasmatic nucleus and neuroendocrine pathways, including the sympathetic nervous system, hypothalamic-pituitary-adrenal axis, and pineal melatonin signaling. These pathways modulate daily variation in adrenaline, cortisol, and melatonin, which may influence NK-cell redistribution and effector outputs. Candidate molecular pathways include IL-15/CD122 signaling, the PDK1–AKT–mTOR–NFIL3/E4BP4 axis, PER1/PER2-associated cytolytic programs, and STRA13-linked regulation of NKG2D ligand-related signaling. Together, these mechanisms may contribute to phase-dependent variation in NK-cell number, cytotoxicity, cytokine production, and immune-surveillance-related functions. The figure was created with BioGDP.com (114).

Overall, the peripheral immune system exhibits a daily dynamic fluctuation pattern, with both the counts and cytotoxic activity of NK cells exhibiting pronounced circadian rhythms closely linked to hormonal and neuroendocrine regulation within the body (15) (**Figure 1**). Research indicates that as “stress-responsive leukocytes,” NK cells exhibit a rapid elevation in their proportion within the peripheral blood following adrenaline stimulation during the morning hours. In healthy male individuals, this peak typically manifests at approximately 08:30 (16, 17). The proportion of NK cells in the morning is significantly higher than that in the afternoon, further demonstrating the pivotal role of hormonal regulation in the distribution of peripheral immune cells (16). In macaque models, monitoring of CD16^+^ NK cells revealed a reduction in their proportion during the dark phase, which is subsequently followed by a substantial rebound occurring between 08:00 and 12:00. This pattern of variation exhibits a strong correlation with the diurnal fluctuations in plasma cortisol concentrations (18). In domestic pig models, NK cell numbers also peaked during daytime hours, which mirrors the observations reported in humans and macaques (19, 20). Concurrently, diurnal peaks and troughs in cortisol secretion directly influence NK-cell functional status. Both the NK-cell proportion and their cytotoxic activity follow a phase-dependent pattern, rather than a uniformly positive correlation; around the endogenous cortisol peak, the association can invert before turning positive again (21). Furthermore, multi-day rhythm studies have uncovered synchronized variations between hormone levels and NK cell proportions, indicating the endocrine system plays a crucial regulatory role in the maintenance of immune homeostasis (22). Collectively, these findings support a temporal association between endocrine rhythms and NK-cell quantity or function under physiological conditions. Nevertheless, the exact phase, amplitude, and direction of these rhythms differ across humans and animal models, and may also depend on whether cell counts, cytotoxicity, cytokine production, or receptor expression is measured. Thus, this section establishes the physiological pattern of NK-cell rhythmicity, whereas the underlying neuroendocrine pathways are discussed in greater detail below.

### 2.2 Photoperiod, artificial light exposure, and circadian desynchronization

In domestic pigs and inbred mouse models, investigations have demonstrated that the circadian rhythms of peripheral immune cells exhibit enhanced prominence under short-day photoperiod conditions. Specifically, under short-day conditions, the rhythmic amplitude of total leukocytes, NK cells, and T cells is elevated, and the peak cell counts of these cell populations occur earlier relative to the light-onset time. Furthermore, the rhythmic modulation of plasma cortisol concentrations exhibits increased prominence (23). In inbred mice, although reproductive function remains unaffected by photoperiod, immune phenotypes in the blood still undergo alterations in response to light exposure—for instance, NK cell activity is enhanced during the nighttime phase (24). These findings support the possibility that photoperiod may influence immune rhythmicity through endocrine signaling and dynamic redistribution of immune cells.

Beyond seasonal photoperiod, prolonged artificial light exposure may also induce broader circadian desynchronization. Animal studies have shown that continuous or long-duration light exposure can disturb central and peripheral clock-gene rhythms, including Bmal1-, Clock-, Per-, Cry-, and Aanat-related oscillations, and can affect endocrine-sensitive tissues through pathways such as Akt/FoxO1 and PKC-α/Akt (25–27). Other light-disruption models further indicate changes in corticosterone levels, inflammatory responses, spleen clock-gene expression, and cell-mediated immunity (28, 29). Although these studies did not directly establish artificial-light-induced NK-cell dysfunction, they broaden the mechanistic context by showing that abnormal light exposure can affect neuroendocrine and immune-associated pathways that may indirectly shape NK-cell rhythmicity.

### 2.3 Seasonal, age, and sex effects

In addition to intrinsic clock mechanisms and hormonal modulation, seasonal variation, aging, and sex-related differences may also modify NK-cell circadian patterns. Seasonal investigations have demonstrated that the circadian rhythm amplitude of NK cells and other T cell subsets in healthy male individuals displays notable seasonal variations. For instance, rhythms are pronounced during certain months while tending toward flatter patterns in others (17). As age advances, the counts of T cells decrease significantly. In contrast, the numbers of NK cells, the functional responsiveness of specific T cell subsets, and the activity of monocytes are notably elevated in elderly individuals—this phenomenon implies the existence of an immunological compensatory mechanism in response to the substantial reduction in T cell numbers (30). In female individuals, fluctuations in progesterone levels throughout the menstrual cycle elicit corresponding alterations in sleep architecture and immune function. The high-progesterone phase delays the onset of slow-wave sleep and postpones the decline in NK cell activity, whereas the low-progesterone phase presents an early activity decline pattern that is comparable to that observed in male individuals (31). Furthermore, the interindividual variability in NK cell activity observed in stress experiments reflects the regulatory role of sex-related factors in the temporal characteristics of immune responses (32).

### 2.4 Exercise timing

Studies conducted on male participants have demonstrated that performing two exercise sessions on the same day leads to a significant elevation in total white blood cell count and neutrophil count, but the enhancement of NK cell activity primarily depends on the exercise timing: afternoon exercise induces a more substantial increase in NK cell activity compared to morning exercise (33). In studies involving women, although baseline counts for most lymphocyte subsets (T cells, B cells, NK cells) varied across different time points, their overall response pattern to exercise—characterized by an initial increase followed by a subsequent decrease—remained consistent. Furthermore, female participants displayed higher baseline levels of NK cells as well as a more robust NK cell response to exercise during the morning hours (34).

Taken together, these findings suggest that exercise-induced NK-cell responses are shaped by circadian timing, sex, and baseline immune state. However, current evidence does not establish a universally optimal exercise window for enhancing NK-cell function. A more cautious interpretation is that exercise timing should be treated as a biological variable in future studies of NK-cell redistribution, cytotoxicity, and recovery dynamics, rather than as an immediate basis for generalized lifestyle or clinical recommendations.

## 3 NK cells in pathological conditions and circadian rhythms

### 3.1 Tumor

**Figure 2:**
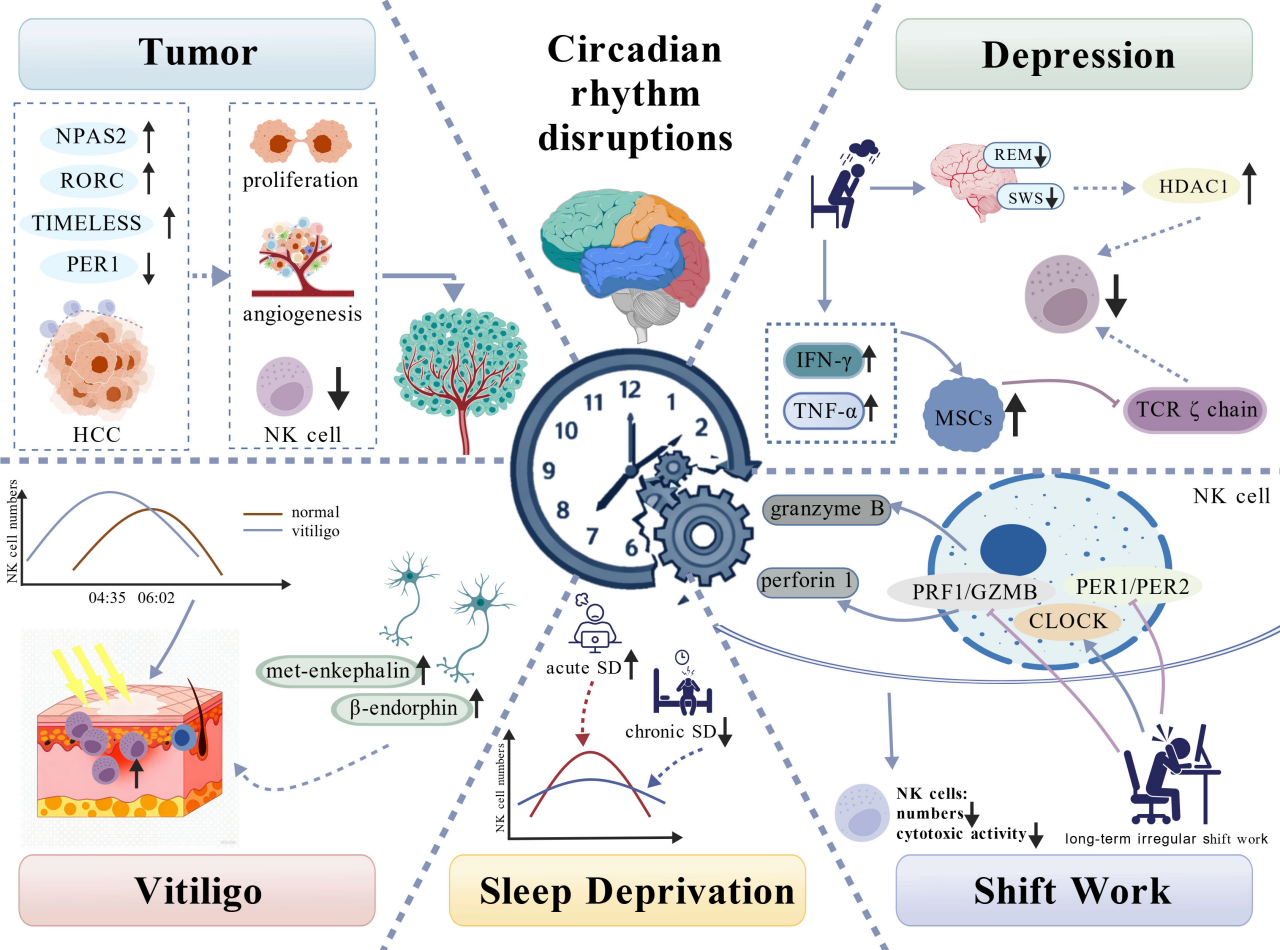
Circadian disruption and NK-cell alterations under pathological and occupational conditions. In tumors such as hepatocellular carcinoma, altered expression of core clock-related genes, including NPAS2, RORC, TIMELESS, and PER1, has been associated with tumor proliferation, angiogenesis, NK-cell-related immune signatures, and changes in the tumor immune microenvironment. In depression, altered sleep architecture, HDAC1-related regulatory changes, systemic inflammatory activation, and TCR ζ-chain downregulation may accompany impaired NK-cell rhythmicity or function. In vitiligo, altered NK-cell rhythmicity has been reported, including an advanced activity peak, together with disrupted neuropeptide secretion involving met-enkephalin and β-endorphin. Sleep deprivation shows exposure-duration-dependent effects on NK-cell readouts, with acute sleep loss and chronic or repeated sleep disruption producing different effects on NK-cell activity, number, or functional stability. Long-term irregular shift work has been associated with reduced NK-cell number and cytotoxic activity, potentially involving altered clock-gene expression and NK-related transcriptional programs. Across these conditions, NK-cell circadian alterations should be interpreted as context-dependent associations rather than uniform causal mechanisms. The figure was created with BioGDP.com (114).

Circadian rhythm disruption is frequently observed in patients with diverse tumor types and has been associated with altered NK-cell number, activity, clock-gene expression, and clinical outcomes (**Figure 2**). However, in most human cancer studies, these associations do not establish whether circadian disruption is a driver of NK-cell dysfunction, a consequence of tumor progression, or a parallel marker of systemic disease burden. In patients with metastatic breast cancer, aberrant cortisol circadian rhythms are often significantly correlated with reduced NK cell counts and impaired functional activity. This rhythmic aberration typically presents as a decreased rhythm amplitude or a “flattened” rhythmic pattern, which serves as an indicator of poor clinical prognosis (35). Studies on lung cancer patients reveal altered 24-hour mean levels of various lymphocyte subsets, such as decreased CD8^+^ T cells and increased CD16^+^ NK cells. Crucially, however, the circadian rhythmicity and peak timing of these cells are often preserved. These findings suggest that, in these lung cancer cohorts, cancer-associated immune alterations may involve changes in rhythm amplitude or 24-hour mean levels while peak timing can remain partly preserved, rather than indicating a complete loss of circadian organization (36–38). Subsequent animal studies have demonstrated that the circadian rhythmicity of NK cells in rats exhibits distinct organ specificity: during the dark phase, the percentage of NK cells in the spleen increases significantly, resulting in enhanced NK cell cytotoxicity, whereas the functional activity of NK cells in the peripheral blood decreases substantially concurrently. This rhythmic fluctuation was associated with enhanced short-term clearance of lung tumor cells; however, it did not translate into a significant long-term reduction in lung metastasis burden (39). These findings illustrate an important translational caveat: phase-dependent NK-cell activity may influence short-term tumor-cell clearance in experimental systems, but this does not necessarily predict long-term metastatic outcome or therapeutic benefit.

Furthermore, aberrant expression of core clock genes is also closely correlated with NK cell activity and tumor progression. In hepatocellular carcinoma (HCC), dysregulation of core circadian clock genes (such as NPAS2, PER1, RORC, NR1D1, and TIMELESS) has been associated with NK-cell-related immune signatures, changes in the tumor immune microenvironment, and poor prognosis (40). However, such transcriptomic associations do not determine whether clock-gene dysregulation directly impairs NK-cell surveillance or instead reflects broader tumor-intrinsic and microenvironmental changes. Similarly, abnormal expression of circadian rhythm-related genes (such as downregulated expression of PER1, PER2, and CRY2, and upregulated expression of CSNK1E) has been identified in prostate cancer patients, accompanied by a significant reduction in NK cell numbers (41). Clinical evidence in breast cancer has also linked bedtime misalignment or circadian disruption with disease progression, supporting a broader association between circadian organization and cancer outcomes. However, these data do not by themselves establish that the observed clinical associations are mediated directly by NK-cell dysfunction (42). Evidence from renal cell carcinoma further suggests that circadian rhythm genes may be linked to cancer-cell proliferation pathways, including mTOR and AMPK, as well as immune-surveillance-related processes. However, whether these clock-related alterations directly regulate NK-cell activity in human tumors remains to be established (9).

Taken together, cancer-related studies suggest that circadian disruption is associated with altered NK-cell number, activity, and clock-gene expression, but the strength of evidence differs substantially across models. Human studies in breast, lung, liver, and prostate cancer are largely observational and often rely on limited sampling windows, making it difficult to determine whether NK-cell alterations are causes, consequences, or parallel markers of tumor progression. By contrast, animal studies provide more controlled evidence for tissue- and phase-specific NK-cell rhythms, but their findings cannot be directly extrapolated to human tumors without caution. These data therefore support a context-dependent model in which circadian disruption may reshape NK-mediated immune surveillance through changes in rhythm amplitude, tissue distribution, and effector function, rather than through a single uniform pathway.

From a translational perspective, these limitations are particularly important. Current cancer-related evidence does not yet define clinically actionable time windows for monitoring NK-cell function, preventing tumor progression, or scheduling NK-related immunotherapies. Most studies lack repeated circadian sampling, paired assessment of NK-cell number and cytotoxic function, direct measurement of intratumoral NK-cell states, and adequate adjustment for treatment exposure, tumor stage, sleep disruption, stress, and chronotype. Future oncology studies should therefore combine longitudinal circadian profiling with tumor-immune phenotyping and prospective intervention designs before circadian timing can be used to guide cancer prevention or treatment decisions.

### 3.2 Depression and sleep disorders

Depression is a common mental disorder closely associated with abnormal NK cell function and circadian rhythm disruption. Studies have demonstrated that in patients with major depressive disorder, both the quantity of NK cells and their cytotoxic activity display markedly attenuated circadian rhythms—this finding suggests a strong association between depressive states and the rhythmic regulation of the immune system (43). Subsequent research has revealed that this NK cell dysfunction is not exclusively caused by a reduction in NK cell numbers; instead, it is primarily associated with depressive mood, anxiety symptoms, and systemic immune activation (44). The persistent inflammatory state associated with depression has been proposed to promote myeloid suppressor-cell activation and downregulation of the T-cell receptor ζ chain (TCR ζ), which may impair T-cell and NK-cell function in some contexts. This pathway provides a plausible link between depression-associated inflammation and reduced immune surveillance, but current clinical evidence remains largely associative and does not establish a direct causal route from depressive symptoms to NK-cell dysfunction or cancer risk (45, 46). Moreover, abnormal expression of clock-linked/epigenetic regulators (e.g., HDAC1, NFIL3, PRKAA1) is particularly pronounced in patients with depression. These genes not only regulate the body’s circadian rhythms but also directly or indirectly participate in maintaining immune system homeostasis. This suggests that immune dysregulation in depression may be closely linked to disturbances in the intrinsic clock mechanism (47).

Sleep disorders, including insomnia and sleep deprivation, can alter NK-cell function by disturbing circadian organization, but the direction and magnitude of these effects depend strongly on exposure duration and sampling phase. In primary insomnia, reduced NK activity coexists with heightened nocturnal sympathetic drive, whereas in depression no significant correlation between sleep disorders and sympathetic nervous activity has been observed (48). Timing and exposure duration are central to interpreting these findings: acute total sleep deprivation (TSD) during the usual sleep phase can transiently raise NK activity (49), whereas multi-day deprivation and subsequent recovery reduce peripheral NK counts—effects that unfold outside the HPA axis (50). Furthermore, sleep and circadian rhythms exhibit synergistic effects in jointly regulating the dynamic distribution of NK cells: under habitual sleep, NK counts show a sleep-linked rebound that is blunted by sustained wakefulness, further highlighting the cooperative control of sleep and circadian phase (51, 52). Collectively, these findings indicate that the effects of depression and sleep disruption on NK cells are not uniformly suppressive. Acute total sleep deprivation may transiently increase NK cytotoxicity, whereas repeated sleep loss, insomnia, or circadian misalignment tends to reduce NK-cell counts, blunt rhythmic redistribution, or impair functional stability. These apparently divergent results likely reflect differences in exposure duration, sampling phase, neuroendocrine state, and the specific NK-cell endpoint measured. Thus, depression- and sleep-related immune alterations should be interpreted as time-window- and context-dependent changes rather than as a single linear pathway of NK-cell suppression.

### 3.3 Vitiligo

Vitiligo is a disease closely associated with immune dysfunction. Earlier chronobiological studies have reported alterations in NK-cell activity, T-cell subset rhythms, and neuropeptide secretion in patients with vitiligo, suggesting that circadian and neuroimmune dysregulation may accompany the disease state. Previous studies have demonstrated that NK cell activity in vitiligo patients is generally higher than in healthy controls, with this effect being particularly pronounced in patients during the quiescent phase. The circadian rhythm peak of NK cell activity in these patients shifts to 4:35 AM, whereas the peak in healthy individuals typically occurs at 6:02 AM (53, 54). Additionally, significant alterations in the circadian rhythms of CD4^+^ and CD8^+^ T cells were observed in the peripheral blood of vitiligo patients: during the active phase, patients exhibited a reduced proportion of CD4^+^ cells and a loss of circadian rhythm, while CD8^+^ cells showed no clear circadian rhythm across all patients. This suggests that abnormal rhythms in T cell subsets may further exacerbate the instability of immune function in patients. Notably, abnormal neuropeptide levels may play a significant role in the dysfunction of NK cells in vitiligo patients. Research indicates that vitiligo patients exhibit significantly elevated levels of met-enkephalin and β-endorphin secretion compared to healthy individuals, yet their characteristic circadian rhythmic fluctuations are absent (54). These neuropeptide changes may reflect altered neuroimmune regulation and could contribute to the abnormal immune rhythmicity observed in vitiligo, although their causal role in disease pathogenesis remains uncertain.

In summary, vitiligo studies suggest altered temporal patterns of NK-cell activity, T-cell subsets, and neuropeptide secretion. However, the available evidence remains limited and largely associative, and the observed phase shifts do not by themselves establish a causal role for NK-cell rhythmicity in disease onset or progression. These findings are therefore best interpreted as indicators of neuroimmune and circadian dysregulation that may contribute to, or accompany, the immunopathological state of vitiligo.

### 3.4 Other pathological examples

Infection and psychological stress provide additional examples in which altered circadian or neuroimmune states have been associated with changes in NK-cell parameters. For instance, in HIV-infected patients, severe psychological stress was associated with lower NK-cell numbers and activity, suggesting a relationship between stress-related immune dysregulation and weakened immune surveillance markers (55). Negative emotions such as anxiety and worry may themselves disrupt the circadian rhythms of NK cells, manifesting as reduced immune regulatory capacity in response to stress (56). Notably, even under severe immune dysregulation like HIV infection, T- and B-cell rhythms can be disrupted while NK rhythms remain preserved, highlighting that rhythmic collapse is not universal and depends on readouts and disease context (57). Furthermore, circadian rhythm alterations in metabolic disorders and neurodegenerative diseases correlate with NK cell dysfunction. For instance, individuals with type 2 diabetes exhibit pronounced sleep disturbances and circadian rhythm disruption, accompanied by abnormally elevated NK cell counts and activity, suggesting rhythm disruption may contribute to chronic inflammatory processes in the disease (58). Patients with Alzheimer’s disease exhibit elevated nocturnal cortisol and inflammatory cytokine levels, similarly accompanied by reduced NK cell activity and circadian rhythm disruption, suggesting that circadian dysregulation may exacerbate immune imbalance (59). Heavy drinkers exhibit reduced deep sleep and increased REM sleep, accompanied by elevated IL-6 levels and decreased NK cell activity, indicating impaired immune regulation. Sleep disturbances may further exacerbate this effect (60). Animal studies indicate that chronic ethanol exposure disrupts circadian expression of NK-cell clock genes and cytolytic mediators, including Per2, Bmal1, perforin, granzyme B, and IFN-γ, thereby weakening NK-cell cytotoxic activity (61). Separately, chronic shift-lag has been shown to alter NK-cell clock rhythms and promote lung tumor growth in rats (62). These pathological examples highlight a broader interpretive challenge. Across infection, psychological stress, metabolic disease, neurodegeneration, and alcohol exposure, NK-cell alterations are measured with different endpoints, including cell counts, cytotoxicity, cytokine production, and clock-gene expression. Some studies report reduced NK activity, whereas others show preserved rhythmicity or even elevated NK-cell counts under chronic inflammatory conditions. Such differences suggest that circadian disruption does not produce a uniform NK-cell phenotype across diseases. Instead, disease-specific inflammation, neuroendocrine tone, tissue compartment, and sampling time may determine whether NK-cell rhythms are shifted, flattened, preserved, or functionally uncoupled from cytotoxic output.

## 4 Shift work and NK cell function

A healthy circadian rhythm is essential for maintaining homeostasis and ensuring efficient immune system function within the body. Disruptions to this rhythm, particularly those caused by irregular or excessive shift work, have been increasingly implicated in studies as potentially impairing NK cell function, thereby elevating disease susceptibility. Studies indicate that prolonged irregular or excessive shift work leads to abnormal expression of circadian rhythm genes in the human body. Specifically, key circadian genes such as Per1 and Per2 are downregulated, while the CLOCK gene is upregulated. This molecular-level imbalance has been linked to reduced NK-cell number and cytotoxic activity, although the extent of this effect may depend on exposure duration, circadian phase, and the contribution of fatigue or sleep debt (63). Studies simulating night shift work in humans have revealed that prolonged night-shift patterns reduce the amplitude of circadian oscillations in the peripheral blood transcriptome and affect NK-related transcriptional programs. These findings support a phase-sensitive association between circadian misalignment and NK-cell regulation, rather than a uniform effect of night work across all settings (64).

Early field studies indicated that simple shift changes alone may not significantly alter NK cell activity or circadian cortisol secretion patterns (65). Subsequent work, however, points to a dose–response with irregular or high-load schedules: emergency physicians and female nurses show depressed NK activity, and fatigue severity tracks the decrement (66, 67). Furthermore, Japanese workplace studies indicate that prolonged overtime or excessive fatigue is associated with lower NK-cell counts, suggesting that chronic excessive or irregular shift work may be associated with weakened NK-cell immune surveillance, although fatigue, sleep debt, psychosocial stress, and sampling time remain important confounding factors in occupational studies (68). Laboratory night-shift simulations and animal models provide complementary lines of evidence: simulated night shift dampens NK-related transcriptional programs, including Jun/AP-1 and STAT pathways (64), whereas animal models show CD122 downregulation with reduced cytotoxicity (8). Thus, the impact of shift work hinges less on “shift” per se than on misalignment magnitude and accumulated fatigue.

In summary, the shift-work literature supports an association between circadian misalignment and altered NK-cell parameters, but the interpretation depends strongly on study design. Field studies in occupational cohorts are valuable for ecological relevance, yet they are often influenced by workload, fatigue, sleep debt, psychosocial stress, and individual chronotype. Laboratory night-shift simulations provide tighter control over light exposure, sleep timing, and sampling phase, whereas animal models allow mechanistic testing of clock-gene and cytokine-receptor pathways. Considered together, these findings suggest that NK-cell impairment is more likely to emerge when shift work involves sustained misalignment, irregular schedules, or accumulated fatigue, rather than from shift status alone.

## 5 Potential molecular mechanisms and signaling pathways

Having summarized physiological, disease-associated, and occupational patterns, this section focuses on candidate mechanisms that may explain time-dependent NK-cell variation. To avoid reiterating descriptive findings, we separate cell-intrinsic clock-gene pathways from systemic neuroendocrine inputs and emphasize the evidence base and limitations of each.

### 5.1 Regulation of clock genes in NK cell development and function

Most direct evidence linking clock genes to NK-cell development or cytotoxic function derives from murine models, genetic perturbation experiments, or *in vitro* systems. These studies are valuable for identifying candidate regulatory pathways, but their findings should not be interpreted as direct proof of equivalent mechanisms in human NK-cell biology. Human evidence remains more limited and often reflects associations between clock-gene expression, endocrine timing, immune-cell phenotype, or disease status. Therefore, the molecular pathways discussed below should be viewed as mechanistic candidates whose relevance to human circadian immunoregulation requires further validation.

#### 5.1.1 NFIL3 (E4BP4) and NK cell generation

NFIL3 (also known as E4BP4) initially gained attention for regulating circadian rhythms in the chicken pineal gland. However, phenotypic analyses conducted in four independent laboratories using E4bp4 (-/-) mice revealed its critical role across multiple hematopoietic lineages. This includes regulating NK cell and CD8α(+) conventional dendritic cell development, macrophage activation, CD4(+) T cell response polarization, and B cell class switching toward IgE production (69). Regarding NK cell generation, NFIL3 not only functions as a circadian rhythm-associated protein but also influences early NK cell differentiation and maturation by interacting with the PDK1-mTOR-NFIL3-CD122 signaling axis (involving PIP3, AKT, mTOR, PTEN, and PDK1). PDK1 deficiency attenuates IL-15-induced mTOR activation and NFIL3 upregulation, leading to reduced NK cell numbers and impaired antitumor activity. Exogenous NFIL3 expression or bypassing mTOR activation partially rescues this defect, suggesting that PDK1-mTOR-E4BP4-CD122 constitutes a critical positive feedback pathway sustaining NK cell development and function (70). Additionally, NFIL3 acts as an inhibitor of CREB-induced gene expression, binding to numerous gene promoter regions and cooperating with other transcription factors (such as C/EBP) to regulate diverse physiological processes, including neurite outgrowth and cell survival (71). Collectively, these studies identify NFIL3/E4BP4 as an important candidate node connecting clock-associated transcriptional regulation with NK-cell development and IL-15 responsiveness, particularly in experimental mouse systems. However, the extent to which this pathway governs mature human NK-cell cytotoxicity or circadian variation in clinical settings remains incompletely defined. Further human-focused studies are needed to determine whether NFIL3 functions as a conserved regulator of NK-cell timing, differentiation, or effector output across physiological and pathological contexts.

#### 5.1.2 Bidirectional regulation of the PER1/PER2-immune axis

The core clock gene PER2 has also been demonstrated to exhibit bidirectional regulatory interactions with immune function. Relevant studies indicate that even under constant darkness conditions, molecular clock genes such as Per2, Clock, and Bmal1 in NK cells continue to oscillate in a 24-hour rhythm alongside cytotoxic factors (e.g., granzyme B, perforin, IFN-γ, and TNF-α). Downregulating Per2 via RNA interference significantly impairs NK cell lytic activity (72, 73). Meanwhile, Per2 knockout mice exhibited enhanced resistance to LPS-induced endotoxin shock, yet demonstrated functional defects in NK and NKT cells within the spleen alongside reduced IFN-γ secretion. This finding provides the first molecular-level evidence of a direct link between Per2 and innate immune defense (74). Furthermore, murine studies suggest that Per2 contributes to the temporal regulation of NK-cell function in bone marrow, in part by modulating the circadian expression of cytotoxicity-associated receptor genes such as Ly49C and Nkg2d. This circadian variation arises not from diurnal differences in NK, NKT, or T cell numbers, but from gene expression driven by the intrinsic biological clock. These findings support the possibility that Ly49C and Nkg2d act as clock-responsive effector-associated genes in this murine context, although their relative contribution to human NK-cell cytotoxicity remains to be clarified (75). Notably, in tumor models, sarcoma cells overexpressing Per2 exhibited heightened sensitivity to NK cell-mediated killing and showed reduced *in vivo* growth, suggesting that PER2 may influence tumor immune surveillance under experimental conditions (76). Conversely, reduced PER2 expression correlates closely with decreased NK cell numbers and functional suppression within the tumor microenvironment, particularly evident in patients with malignant tumors. This association suggests that clock-gene abnormalities may be linked to immune alterations within the tumor microenvironment, but it does not establish a direct causal mechanism in human tumors (41). Beyond PER2, another core clock gene, PER1, is equally indispensable for maintaining immune system homeostasis. Research reveals that Per1 gene mutations (Per1^-^/^-^) disrupt the circadian rhythms of interferon-gamma and cytotoxic factors in NK cells, accompanied by altered expression patterns of other clock genes (such as Bmal1 and Per2). Under constant darkness, mice still maintained spontaneous running cycles on the wheel, though significantly shorter in duration, suggesting Per1 is equally important for coupling the peripheral molecular clock with immune function (77). The abnormal NK cell rhythms caused by this gene deletion further substantiate the close relationship between core clock genes and innate immune responses.

Taken together, the NFIL3/E4BP4 and PER1/2 findings support complementary, but not identical, links between the molecular clock and NK-cell biology. NFIL3-related evidence mainly points to early NK-cell differentiation and IL-15/CD122 responsiveness (70), whereas PER1/2-related studies implicate rhythmic expression of cytotoxic mediators and receptor programs in experimental systems (72, 74–77). Evidence from glucocorticoid-timing studies and laboratory night-shift simulations further suggests that endocrine timing and circadian misalignment can reshape NK-related phenotypes or transcriptional programs, but these findings arise from different experimental and clinical contexts (64, 78, 79). These pathways should therefore be interpreted as candidate mechanisms operating at different levels of NK-cell regulation, rather than as a single linear PER–NFIL3 axis. Their relevance to mature human NK-cell cytotoxic rhythms remains insufficiently established and will require timed human sampling, perturbation-based designs, and paired count–function readouts.

#### 5.1.3 Regulatory role of STRA13

The clock gene STRA13 has also been implicated in the temporal regulation of NK-cell activation-related signals. In experimental systems, circadian transcriptional regulation involving STRA13 influences the expression of NKG2D receptor ligands, which are important activation-related signals for NK cells (80). These findings suggest that STRA13 may contribute to the temporal regulation of NK-cell activation-related signals. However, as with PER- and NFIL3-related pathways, additional studies are needed to determine whether this regulatory mechanism operates similarly in human NK cells under physiological or disease conditions.

### 5.2 Neuroendocrine-immune interactions

Neuroendocrine pathways provide systemic timing cues that help coordinate NK-cell trafficking and effector function. To avoid overlap with the physiological descriptions above, this section focuses on three major regulatory routes: the pineal–melatonin axis, sympathetic nervous system signaling, and HPA-axis/glucocorticoid signaling. These pathways interact with each other, but they are discussed separately to clarify their main evidence base and avoid conflating endocrine association with causal mechanism.

#### 5.2.1 Melatonin and other circadian regulatory molecules

Melatonin is a major pineal-derived circadian hormone and provides one route through which environmental light–dark information can influence immune regulation (81, 82). Its secretion typically peaks at night and is closely linked to sleep–wake timing (83). In experimental studies, rhythmic NK-cell cytotoxicity and the expression of effector molecules such as perforin and IFN-γ have been observed alongside daily melatonin and corticosteroid rhythms (72). These findings suggest that melatonin may contribute to the temporal organization of NK-cell activity, although its effects are difficult to separate from other concurrent endocrine and behavioral signals. Melatonin-related pathways may also intersect with immune metabolism. Tryptophan metabolites such as kynurenine and indole-3-pyruvate can activate the aryl hydrocarbon receptor and affect antiviral or antitumor immune responses involving NK cells and CD8^+^ T cells (81, 84). In aging and circadian disruption, altered melatonin signaling has been associated with broader immune changes, including reduced NK-cell function or altered lymphoid responses (85, 86). However, these observations should be interpreted as part of a broader neuroendocrine-immune network rather than as evidence that melatonin alone determines NK-cell rhythmicity.

#### 5.2.2 Stress duration, sympathetic nervous system regulation, and phase dependence

Research indicates that the sympathetic nervous system transmits circadian rhythm signals to immune cells, particularly NK cells, through rhythmic release of norepinephrine. Experiments reveal that norepinephrine input within the spleen exhibits distinct circadian rhythmicity. This input synchronizes the molecular clocks of NK cells and other splenic cells, thereby regulating their cytokine secretion and cytolytic factor expression (87). Additionally, NK cells express high levels of adrenergic receptors on their surfaces, rendering them particularly sensitive to neurotransmitters released by the sympathetic nervous system. This results in fluctuations in both their numbers and activity throughout the day. For instance, some studies have found that morning adrenergic stimulation can elevate NK cell counts to peak levels, suggesting that sympathetic nervous system input participates in regulating the dynamic distribution of immune cells (88).

Under acute stress conditions, the sympathetic nervous system rapidly releases neurotransmitters such as norepinephrine, whose signals exert pronounced circadian rhythmic effects on the immune system. The neural signals triggered by acute stress stimuli produce varying effects at different times: morning stress often significantly increases NK cell activity and numbers, while other times of day may show varying degrees of suppression or reduced responsiveness (61, 89–91).

Additionally, chronic stress-induced immune dysfunction may exacerbate the immune aging process through IL-6-mediated disruption of metallothionein (MT) homeostasis. Persistently elevated IL-6 under prolonged stress may increase zinc-bound metallothionein (MT), reducing zinc bioavailability and thereby impairing the functional plasticity of NK cells and hepatic NKT γδ cells (92). Research indicates that this zinc homeostasis imbalance can be partially restored in young and extremely aged animals through circadian rhythm remodeling, thereby maintaining NK cell immune surveillance function. However, middle-aged and elderly animals lack this effective MT remodeling capacity, manifesting as significantly impaired NK cell function and reduced host defense against viruses and tumors (93).

Taken together, stress-related effects on NK cells should be interpreted through both circadian phase and exposure duration. Short-term sympathetic activation can redistribute NK cells and may transiently increase NK-cell activity, particularly when the stressor occurs at a permissive circadian phase or when baseline cytotoxicity is low. In contrast, prolonged stress or chronic inflammatory activation may impair NK-cell plasticity through sustained cytokine signaling and altered metallothionein-related zinc homeostasis. Thus, stress is not uniformly immunostimulatory or immunosuppressive; its effects on NK cells depend on timing, duration, neuroendocrine state, age-related immune plasticity, and the functional endpoint examined.

#### 5.2.3 HPA axis regulation and glucocorticoid rhythm

The HPA axis represents another major endocrine pathway linking circadian timing, stress physiology, and NK-cell regulation. As noted above, cortisol shows a robust daily rhythm, and NK-cell redistribution or cytotoxicity can vary in relation to this endocrine phase (11, 94–96). Rather than repeating the descriptive rhythm patterns, this section focuses on the potential mechanisms by which glucocorticoid signaling may shape NK-cell phenotype and function. Mechanistically, glucocorticoids act through intracellular glucocorticoid receptors and can suppress NK-cell development or reduce cytokine production, including IFN-γ, in experimental models (97, 98).

Timing-sensitive endocrine interventions further suggest that NK-cell responses may depend on the phase of HPA-axis activation: ACTH administration has been reported to enhance NK activity more strongly in the morning than at night, and glucocorticoid replacement schedules can alter clock-controlled immune-cell markers such as LAMP-1/CD107a and NKG2D/KLRK1 (78, 79, 99). These findings support the biological relevance of glucocorticoid timing, but they should not be interpreted as sufficient evidence for changing glucocorticoid dosing schedules to optimize NK-cell function outside disease-specific clinical indications or controlled trials.

## 6 Future research directions and emerging translational perspectives

Circadian timing offers a useful lens for understanding why NK-cell phenotypes vary across studies and clinical settings, but its use as an intervention principle remains preliminary. Current evidence supports time-of-day variation in NK-cell number, cytotoxicity, receptor expression, and transcriptional programs, as well as modulation by sleep, shift work, endocrine signals, and experimental clock-gene perturbation. However, translating these observations into cytokine scheduling, glucocorticoid timing, melatonin or nutritional supplementation, clock-pathway pharmacology, or exercise and light-timing strategies will require prospective studies with defined circadian phases, paired immune endpoints, and clinically meaningful outcomes. These approaches are therefore discussed below as emerging research directions rather than as ready-to-use clinical strategies.

### 6.1 Methodological improvements

#### 6.1.1 Longitudinal and large-scale population studies

Existing studies are largely limited to small sample sizes and cross-sectional designs, making it difficult to track the dynamic temporal changes in individual circadian rhythms and NK cell function over time. At a minimum, new work should report the sampling phase (ZT/CT or local clock time), sleep–wake state, and recent exposures, and should pair count and function endpoints (e.g., counts with CD107a/IFN-γ) to reconcile directionally discordant readouts. Prospective, adequately powered cohorts with high-frequency longitudinal sampling and continuous monitoring are needed to characterize the phase, amplitude, and stability of NK rhythms across diverse physiological and disease contexts. Expanding this research methodology will provide robust data support for a deeper understanding of the relationship between NK cells and circadian rhythms.

#### 6.1.2 High-dimensional, single-cell, and temporal multi-omics profiling

With the advancement of single-cell sequencing, high-dimensional cytometry, spatial profiling, and multi-omics integration technologies, future studies can move beyond bulk NK-cell counts and conventional cytotoxicity assays (100). Recent studies illustrate the value of these approaches: single-cell transcriptomic analysis has been used to characterize circadian rhythm disruption in lung adenocarcinoma and its association with tumor-cell states, drug resistance, and immune microenvironment features (101), while circadian time-prediction methods applied to bulk and single-cell pseudobulk data have revealed cell-type-specific rhythmic programs in dermal immune cells (102). Emerging single-cell atlases across day-night cycles further show that immune-cell populations and transcriptional states can vary by sampling time, although such datasets still require careful validation before direct extrapolation to human NK-cell biology (103).

For NK-cell research, high-dimensional flow cytometry, mass cytometry, single-cell RNA sequencing, and spatial transcriptomics could help distinguish whether circadian variation reflects changes in cell abundance, subset composition, activation state, receptor expression, cytotoxic molecules, or tissue localization. These approaches should be paired with rigorous temporal design, including clearly reported sampling phase, repeated sampling where feasible, and paired count–function endpoints. Without such temporal standardization, high-dimensional datasets may capture technical or compositional variation rather than true circadian regulation of NK-cell states.

### 6.2 Potential timing-based strategies requiring clinical validation

#### 6.2.1 Adjusting work schedules and sleep management

For shift workers, schedule design and sleep management remain practical areas for future occupational health research. More regular shift rotations, adequate recovery time, and strategies to reduce accumulated fatigue may help limit circadian misalignment, but their effects on NK-cell rhythms and function require more direct evaluation (104, 105). Wearable devices and sleep-monitoring tools may also provide useful information on sleep timing, sleep duration, and individual rhythm stability. However, such tools should currently be regarded as supportive monitoring approaches rather than validated interventions for improving NK-cell function or immune surveillance (106).

#### 6.2.2 Nutrition and supplement interventions

Nutritional and supplement-based approaches have also been explored in shift workers and other populations, including probiotics, fermented dairy products, vitamin D, and melatonin (107–110). Some preliminary studies suggest possible changes in NK-cell activity or immune parameters, but the evidence remains limited, heterogeneous, and not sufficient to support routine supplementation for NK-cell optimization. Melatonin is particularly relevant to circadian biology, yet its use for improving NK-cell function in shift workers or patients with circadian disruption should be considered investigational rather than clinically established. Future randomized controlled trials should define the target population, dosing schedule, circadian phase, safety profile, and paired immune endpoints before such interventions can be recommended.

#### 6.2.3 Pharmacological targets and immunomodulatory hypotheses

Clock-related pathways such as NFIL3, PER1, PER2, and PI3K/AKT/mTOR provide useful mechanistic entry points for understanding how circadian timing may shape NK-cell development, responsiveness, and effector function (70, 72, 75, 77). However, pharmacologic manipulation of these pathways for the purpose of correcting NK-cell dysfunction remains highly preliminary. Clock genes and metabolic signaling pathways are pleiotropic and tissue-wide, so interventions aimed at these nodes may have broad systemic effects beyond NK cells. At this stage, these pathways are best viewed as mechanistic hypotheses and potential research targets. Any future pharmacological strategy would require careful validation of target specificity, timing, dose, safety, and immune efficacy before clinical translation can be considered.

### 6.3 Interdisciplinary translation

#### 6.3.1 From basic research to public health

Current research remains concentrated at the cellular, molecular, and observational levels, whereas translation into public health practice is still limited. Future progress will require collaboration across immunology, occupational medicine, sleep science, nutrition, and psychology to design studies that connect circadian exposure, NK-cell endpoints, and clinically meaningful outcomes. Such interdisciplinary work may provide an evidence base for future guidance for shift workers, individuals with insomnia, or populations exposed to chronic circadian disruption. However, broad public health recommendations should await prospective studies that demonstrate reproducible benefits on immune function and health outcomes (111).

#### 6.3.2 Applications in oncology and other major diseases

Circadian regulation of NK-cell biology may be relevant to oncology, but its clinical use remains at an early and largely exploratory stage. Time-of-day effects have been explored in relation to cancer treatment outcomes, and NK-based strategies such as CAR-NK therapy provide a conceptual setting in which immune timing may eventually become relevant (112, 113). However, current evidence is not sufficient to recommend circadian-based scheduling of immunotherapy, CAR-NK treatment, or combined treatment regimens in routine oncology practice. Future studies should first test whether treatment timing reproducibly affects NK-cell phenotype, intratumoral immune activity, therapeutic efficacy, and toxicity in prospective and adequately controlled clinical settings. At present, circadian-guided oncology should therefore be regarded as a hypothesis-generating research direction rather than an established precision-medicine strategy.

## 7 Summary

This review compiled and summarized the current literature on NK cells and circadian rhythm disruption (**Table 1**). Overall, NK cells show measurable circadian organization in both number and effector function, but the reported direction and magnitude of these rhythms vary across experimental systems and clinical settings. Core clock genes and major neuroendocrine inputs appear to shape the phase and amplitude of NK-cell counts and functional outputs, although their relative contributions differ across models and readouts. Findings that appear inconsistent can often be partly explained by differences in sampling phase, exposure duration, species, tissue compartment, and functional readout: acute sleep loss may transiently increase NK activity, whereas multi-day deprivation or circadian misalignment is more often associated with reduced counts or impaired functional stability. Across cancer, depression, vitiligo, and infection, alterations are heterogeneous and may appear as phase shifts, amplitude flattening, preserved rhythmicity, or uncoupling between cell number and effector function. For interpretation, NK-cell “count” and “function” should therefore be evaluated together, and the sampling phase should be explicitly reported. This phase- and context-dependent framework is particularly important for interpreting sleep deprivation, stress exposure, exercise timing, and shift work, where acute and chronic perturbations can produce different or even opposite NK-cell phenotypes.

**Table 1:** Evidence summary of circadian regulation of NK-cell biology across physiological, pathological, mechanistic, and translational contexts.

Category	Year / PMID	Population / model	Study design	Main NK-related finding	Evidence type / key limitation
Physiological rhythmicity	1988 / 3257498	Healthy adults (male-predominant)	Longitudinal repeated-measures study	Circulating NK-cell subsets showed daily and seasonal variation, with a morning peak reported in healthy males.	Human observational rhythm study; male-predominant cohort and older sampling design.
	1995 / 8788239	Older vs young adults	Cross-sectional study with time-series sampling	Aged group had increased activated NK cells and monocyte-derived cytokines, relative to younger controls	Observational; age-related confounders (comorbidities, inflammation).
	1995 / 10607154	Healthy young women	Observational/experimental study	High-progesterone phase delayed the nocturnal decline in NK cell activity compared to low-progesterone phase.	Human observational evidence; sex hormone effects may be confounded by other menstrual cycle factors.
	1997 / 9021865	Healthy adults	Time-series observational study	NK-cell proportion and cytotoxicity varied in temporal association with cortisol rhythm.	Human observational evidence; association does not prove direct endocrine control.
	1997 / 9409657	Human peripheral leukocytes	Comprehensive rhythm analysis	NK cells (daytime-rhythmic cells) expressed high density of adrenergic receptors; morning adrenergic stimulation elevated NK cell counts.	Human observational evidence; receptor expression data does not confirm functional causality.
	2001 / 11745421	344 rats	Controlled animal experiment with circadian sampling	During the dark phase, splenic NK cell percentage and cytotoxicity increased, while blood NK cytotoxicity decreased independent of cell numbers. Lung clearance of tumor cells was enhanced, but long-term metastasis formation was unaffected.	Animal mechanistic evidence; findings are organ-specific and short-term effects did not translate to long-term metastatic outcome.
	2002 / 12426466	Macaque monkeys	Time-series observational study	CD16^+^ NK cell percentages decreased during the dark phase (20:00-04:00) and increased in the morning, paralleling cortisol variation.	Animal observational; phase relationship may not directly translate to humans.
	2011 / 22023763	Healthy male adults	Time-series observational study	Midday peaks for NK cells were identified.	Human observational; small sample, male only.
	2018 / 29017838	Domestic pigs	Time-series observational study	NK cell numbers peaked during daytime hours.	Animal observational; no direct functional assessment.
	2019 / 30915069	Domestic pigs	Photoperiod manipulation study	Short-day photoperiod altered leukocyte rhythmic amplitude and phase, including NK cells.	Animal experimental evidence; photoperiod effects in humans require further investigation.
Physiological rhythmicity	2021–2023 / 35399124; 35841738; 37365144; 33937367	Rodent and hamster light-disruption models	Continuous or long-duration artificial light / dim-light-at-night models	Prolonged abnormal light exposure altered central and peripheral clock-gene rhythms, Aanat/Bmal1-related oscillations, endocrine or inflammatory markers, and cell-mediated immunity.	Animal light-disruption evidence; NK-specific endpoints were mostly not assessed, so relevance to NK-cell rhythmicity remains indirect.
	2023 / 37478465	Healthy adults	Prospective cohort with self-controlled design	Morning NK cell proportions were significantly higher than afternoon proportions.	Human prospective evidence; functional activity was not assessed.
Sleep/stress/shift work	1997 / 9127011	Healthy adults	Controlled crossover study	NK cell counts rebounded higher following sleep compared to continuous wakefulness.	Controlled human experiment; short-term observation only.
	1997 / 9001913	Healthy adults	Within-subjects pre-post field study	Simple shift work changes alone did not significantly alter NK cell activity.	Small-scale field study; limited statistical power.
	1997 / 9107628	Healthy adult women	Crossover interventional study	Females had higher baseline NK cell levels and stronger NK cell responses to morning exercise.	Human interventional evidence; sex differences require larger mixed-cohort validation.
	1998 / 9776001	Healthy males	Intraday repeated-measures study	The magnitude of NK cell response to stress varied by time of day, peaking in the afternoon.	Human observational evidence; stress response variability not fully controlled.
	1999 / 10373274	Trait-worry individuals vs healthy adults	Controlled laboratory experiment	High-worry individuals showed blunted NK cell responses to acute stress.	Controlled human experiment; chronic worry effects on long-term NK rhythm remain unclear.
	2000 / 10738860	Healthy adults	Multi-day longitudinal rhythm study	Total sleep deprivation and subsequent recovery sleep reduced peripheral NK cell numbers.	Controlled human study; effects differ from acute partial sleep loss.
	2001 / 11683483	Healthy adults	Controlled crossover sleep-deprivation study	Acute total sleep deprivation was associated with a transient increase in NK-cell activity during the usual sleep phase.	Controlled human experiment; acute exposure only, no long-term follow-up.
	2003 / 12946658	Primary insomnia/ major depression patients vs healthy controls	Case-control observational study	Reduced NK cell function was associated with disrupted sleep and heightened nocturnal sympathetic activity.	Observational; mood, sleep, and medication confounders.
	2008 / 18403864	Japanese emergency physicians	Cross-sectional occupational study	Shift-working physicians showed lower NK-cell activity than non-shift-work groups.	Observational occupational study; workload, fatigue and stress are major unmeasured confounders.
	2011 / 21778660	Female shift-working nurses	Longitudinal cohort study	Shift work was associated with reduced NK-cell function, with fatigue severity tracking the magnitude of the effect.	Longitudinal human evidence; residual confounding by lifestyle factors cannot be excluded.
	2012 / 21766158	Full-time non-shift Japanese employees	Cross-sectional study	Overtime hours were inversely associated with NK cell counts, but not with NK cytotoxicity.	Observational; residual confounding from lifestyle factors.
	2012 / 22308312	Fischer 344 rats	Chronic shift-work simulation	Chronic circadian disruption reduced NK cell cytolytic activity in rats.	Animal experimental evidence; direct human relevance requires validation.
	2018 / 29735673	Healthy adults	Laboratory night-shift simulation	Simulated night shift dampened circadian transcriptomic amplitude and affected NK-related transcriptional programs.	Controlled human simulation; transcriptomic readout rather than direct functional cytotoxic assay.
	2020 / 33185980	Mice	Chronic circadian disruption model	Chronic shift-lag reduced NK-cell cytotoxic activity and CD122 expression in an animal model.	Mechanistic animal evidence; human translation requires clinical validation.
	1995 / 7694902	HIV-infected patients vs healthy adults	Longitudinal cohort study	Severe psychological stress was associated with decreased NK cell numbers and activity in HIV-infected patients.	Observational cohort evidence; disease progression may confound stress effects.
	1995 / 7730652	Rats	Acute stress intervention study	NK cells showed greater stress-induced numerical decrease than T cells among lymphocyte subsets.	Animal experimental evidence; stress response differences between species may exist.
Disease-associated evidence	1989 / 2715406	Vitiligo patients vs healthy adults	Case-control study with repeated measures	Vitiligo patients showed altered NK-cell activity with time-specific differences compared to controls.	Observational; does not establish NK rhythmicity as a disease driver.
	1992 / 1533492	Major depression patients vs healthy controls	Case-control observational study	Major depression was associated with attenuated circadian rhythms in NK cell number and cytotoxicity.	Observational evidence; mood, sleep, inflammation and medication may confound interpretation.
	1992 / 1316387	Vitiligo patients vs healthy adults	Case-control study with circadian analysis	Vitiligo patients had elevated NK cell activity but preserved normal circadian rhythm, accompanied by neuropeptide secretion abnormalities.	Observational evidence; neuroimmune dysregulation is correlational, not causal.
	1993 / 8403073	HIV-infected patients vs healthy adults	Case-control study with circadian analysis	HIV infection disrupted circadian rhythms in T and B cells but not in NK cells.	Observational; reflects early-stage disease, may not apply to AIDS.
	1994 / 7938562	Depressed inpatients vs healthy controls	Case-control observational study	Reduced NK cell activity in major depression reflected systemic immune activation rather than mere changes in NK cell numbers.	Observational evidence; causal pathway between depression and NK dysfunction not established.
	1999 / 10459492	Lung cancer patients vs healthy adults	Cross-sectional study	NK cell counts were significantly increased in early-stage (I-II) lung cancer patients, with preserved circadian rhythm.	Observational; limited causal inference, does not address function.
	2000 / 10861311	Metastatic breast cancer patients	Observational cohort study	Aberrant cortisol circadian rhythms were associated with reduced NK cell counts and function, and poorer prognosis.	Clinical association study; causal NK-mediated pathway not confirmed.
	2003 / 12554818	Alcoholic patients vs healthy adults	Case-control study with serial nocturnal measurements	Decreased NK cell activity was associated with reduced deep sleep and elevated IL-6 in heavy drinkers.	Observational; complex interactions between alcohol, sleep, and immunity.
	2003 / 12797908	NSCLC patients vs healthy elderly controls	Cross-sectional study	NSCLC patients had increased NK cell counts and elevated IL-2 levels compared with controls.	Cross-sectional evidence; disease stage heterogeneity limits generalizability.
	2009 / 19640600	Type 2 diabetes patients vs healthy adults	Case-control observational study	Type 2 diabetes was associated with sleep disturbances, circadian disruption, and abnormally elevated NK cell counts and activity.	Observational evidence; metabolic abnormalities may confound circadian and immune changes.
	2010 / 19728317	Alzheimer's disease (AD) patients with agitation vs healthy adults	Longitudinal observational study	Reduced NK cell activity was associated with circadian disruption and elevated nocturnal cortisol in AD.	Observational; disease-specific, cannot separate from neurodegeneration.
	2012 / 21910027	NSCLC patients vs healthy elderly controls	Cross-sectional circadian profiling	NSCLC patients had higher 24-hour mean CD16^+^ NK cell levels than controls.	Cross-sectional evidence; limited causal inference.
	2014 / 24156520	Metastatic breast cancer patients	Retrospective cohort study	Lower NK cell count was independently associated with shorter disease-free interval.	Retrospective clinical association; marker, not necessarily a mechanism.
	2022 / 35154101	Prostate cancer patients (TCGA cohort)	Retrospective bioinformatic analysis	Dysregulated circadian gene expression was associated with reduced NK cell infiltration in high-risk patients.	Bioinformatic association; requires experimental and clinical validation.
	2022 / 35436363	Human lung adenocarcinoma (LUAD) patients and cell lines	Single-cell transcriptomic analysis with clinical correlation	Circadian rhythm disruption signatures were associated with poor prognosis, drug resistance, and altered NK cell infiltration patterns in LUAD.	Retrospective bioinformatic analysis; functional validation of NK cell-tumor interactions is required.
	2023 / 37969365	Hepatocellular carcinoma (HCC) patients (TCGA and other databases)	Retrospective bioinformatic analysis	A circadian gene signature was associated with poor prognosis and lower NK-cell infiltration scores.	Bioinformatic association; functional validation in patient samples is needed.
	2023 / 37633529	MDD patients vs healthy controls (GEO datasets)	Retrospective bioinformatic analysis	Resting NK cell proportions differed significantly between MDD patients and controls, with HDAC1 showing the strongest correlation.	Bioinformatic evidence; requires experimental validation in human samples.
Mechanistic evidence	1990 / 2092865	Sprague-Dawley rats	Surgical intervention with circadian sampling	Pineal and sympathetic pathway manipulation altered mononuclear-cell rhythmicity involving NK cells.	Animal mechanistic evidence; pathway relevance to human NK rhythms requires validation.
	2004 / 14581485	Stra13-deficient mice	Gene-function study	STRA13 regulated circadian expression of an NKG2D receptor ligand.	Mechanistic animal evidence; direct human NK function remains uncertain.
	2004 / 14978081	Sprague-Dawley male rats	Controlled animal experiment	Ethanol disrupted NK cell circadian rhythms by altering expression of clock genes (Per2, Bmal1) and cytolytic factors (perforin, granzyme B).	Animal mechanistic evidence; human alcohol exposure effects may differ.
	2006 / 16309885	Rat NK cell line (RNK16)	RNAi-based experimental study	Per2 knockdown reduced granzyme B and perforin expression in rat NK cells.	In vitro animal cell evidence; human NK cell clock regulation requires validation.
	2006 / 16861663	Per2-deficient mice vs wild-type controls	Gene-deficiency model study	Per2 knockout mice had functional defects in splenic NK and NKT cells, with reduced IFN-γ secretion.	Mechanistic animal evidence; human Per2 function in NK cells is not fully characterized.
	2007 / 17903270	Young/old/very old mice, centenarians and elderly individuals	Combined animal and human cohort study	IL-6-mediated disruption of zinc-bound Metallothionein homeostasis was linked to impaired NK cell plasticity.	Combined animal-human evidence; causal pathway in humans requires confirmation.
	2008 / 17991807	TLR agonist-treated mice	Experimental study	Chronic TLR activation induced NK-cell immunosuppression through zeta-chain degradation in mice.	Animal model of chronic inflammation; pathogen-free, not natural infection.
	2009 / 20030538	mPer2-deficient mice vs wild-type controls	Gene-deficiency model study	mPer2 knockout downregulated mRNA levels of key NK cytotoxicity regulators (Ly49C, Ly49I, Nkg2d) in mouse bone marrow.	Mechanistic animal evidence; gene expression changes do not confirm functional effects in mature NK cells.
	2010 / 20236181	Murine sarcoma 180 cell lines	Gene-function study	PER2 overexpression in sarcoma cells enhanced their susceptibility to NK cell-mediated killing in vivo.	Animal tumor model evidence; human tumor PER2-NK interaction requires validation.
	2011 / 20816749	Rat spleen and enriched NK cells	Chemical sympathectomy study	Rhythmic splenic norepinephrine input entrained NK cell molecular clock and cytolytic function in rats.	Animal neuroimmune mechanism; human sympathetic regulation of NK cells remains indirect.
	2013 / 23402528	Per1-deficient mice vs wild-type controls	Gene-deficiency model study	Per1 gene modulated immune pathways through NK cell intrinsic clocks in mice.	Mechanistic animal evidence; human Per1 function in NK cells is unclear.
	2015 / 25624444	Conditional PDK1-deficient mice	Basic mechanistic study	PDK1–mTOR–E4BP4 signaling supported IL-15 responsiveness during early NK-cell development.	Mechanistic mouse evidence; mature human NK-cell timing not established.
Methodological / high-dimensional evidence	2024 / 38714698	Multiple human and mouse transcriptomic datasets	Computational method development and validation study	The tauFisher pipeline was developed to infer circadian time from bulk and single-cell pseudobulk transcriptomic data, supporting cell-type-specific rhythmic analysis.	Methodological evidence; NK-specific functional validation remains limited.
Translational evidence	1991 / 1651855	Pigs and peripheral blood lymphocytes	In vivo/in vitro hormone intervention study	Morning ACTH administration enhanced NK cytotoxicity when baseline killing capacity was low.	Animal endocrine intervention; not a clinical dosing recommendation.
	2018 / 29846607	Adrenal insufficiency patients vs healthy controls	Randomized controlled trial (RCT) ancillary study	Glucocorticoid administration timing was associated with immune cell clock gene expression and CD16^+^ NK cell proportions.	Clinical RCT evidence; NK-specific functional impact remains limited.
	2025 / 40003944	Pediatric/adolescent congenital adrenal hyperplasia (CAH) patients	Prospective bi-centric cohort study	Reverse-circadian glucocorticoid treatment altered NK cell phenotypes (reduced CD107a, increased NKp30 expression).	Disease-specific clinical cohort; findings cannot be generalized to healthy populations.

NK, natural killer; HPA, hypothalamic–pituitary–adrenal; PBMC, peripheral blood mononuclear cell; TSD, total sleep deprivation; MDD, major depressive disorder; HCC, hepatocellular carcinoma; NSCLC, non-small cell lung cancer; CAH, congenital adrenal hyperplasia; RCT, randomized controlled trial. Studies are grouped by primary biological or clinical context rather than by publication year. Evidence type and limitations are summarized to distinguish mechanistic animal or in vitro studies, observational human associations, methodological studies, and exploratory translational evidence.

Clock control may connect to NK-cell effector programs through several candidate molecular nodes. Experimental studies implicate the NFIL3–CD122 axis, PER-associated cytotoxic receptor programs, CD122–JAK–STAT5/T-bet signaling, and AP-1/STAT signatures as potential links between circadian timing and NK-cell responsiveness. However, many of these links are derived from animal models or controlled experimental systems, and their relevance to human NK-cell cytotoxic rhythms remains to be validated through longitudinal and perturbation-based studies. To define NK-rhythm phase, amplitude, and stability across settings, the field now needs well-powered longitudinal cohorts that combine high-frequency sampling with continuous monitoring (e.g., actigraphy; salivary cortisol/melatonin). As a minimum reporting standard, studies should include ZT/CT (or clock time), sleep–wake state and recent exposures, and paired count–function endpoints (e.g., counts with CD107a/IFN-γ) to reconcile directionally discordant readouts.

From a translational perspective, the most immediate implication is not to implement NK-focused chronotherapy, but to treat time as a biological variable in study design, data interpretation, and future intervention trials. Timing-based approaches—including glucocorticoid scheduling, cytokine-based immune modulation, light or exercise timing, and fatigue-reduction strategies for shift workers—are biologically plausible but remain insufficiently validated and require prospective testing with clinically relevant endpoints. Until such evidence is available, these approaches should be framed as research priorities rather than established strategies for preserving NK-cell immune surveillance or improving clinical outcomes. Overall, the available literature is best interpreted as evidence for a phase- and context-dependent circadian influence on NK-cell biology, rather than as a uniform mechanistic or clinical framework ready for direct translation.
